# A Strange People

**Published:** 1864-08

**Authors:** 


### A STRANGE PEOPLE.

There are many odd countries in the world, whose inhabitants rejoice in many odd customs; but for the oddest of people, and the queerest of manners, commend us to those islands included in the sovereignty of Japan.

Until a very recent date, no Europeans were permitted to trespass beyond the sacred limits of this most exclusive of empires, nor were any Japanese allowed to quit their native shores. Even now, when you land at Nagasaki, your movements are watched by regular sentries, who report every step you take to their superiors; while to prevent the Japanese themselves from roaming to foreign lands, all their vessels are built after a government model, with open sterns, so that long sea voyages are impossible; and if they exclude us from visiting them, they are in turn equally debarred from visiting us.

They need not be afraid of visitors, from any possibility of being overpowered by numbers; for the thousand and one isles which make up the empire of Japan, contain thirteen thousand densely peopled towns. Jeddo, the capital, seated in the island of Niphon, has a population nearly equal to that of London; and we are told by travellers that the castle in which resides the secular emperor (there are two emperors—one sacred, one secular,) could accomodate forty thousand men. Miako, a city covering twelve square miles, could raise a battalion of fifty-two thousand priests alone; while Osacco, the Birmingham of the empire, could itself send forth an army of eighty thousand.

"You scarcely emerge from one borough," says Kæmpfer, "but you enter another; and you may travel many miles, as it were, in one street, without knowing it to be composed of many villages, save by the different names that were formerly given them, and which they after retained, though joined to one another."

Earthquakes are disastrously frequent in Japan, and are of terribly long duration. One in 1586 lasted, with varying intensity, for forty days. Two hundred thousand perished at Jeddo, during the convulsion of 1703; and a large city was prostrated by that of 1792. It becomes impossible, therefore, for the Japanese architects, to construct lofty piles out of clay and bamboos, and the chimneys of the Manchester factories would be out of place in Niphon. The law restricts the height of a dwelling to six kins, or forty-four feet three inches, and there are few houses which boast of more than one story.

Let us walk into a Japanese house, passing without notice the worthy householder, who sits in a tub of water at the door, performing his ablutions with a refreshing freedom from bashfulness. You notice that the floor is slightly raised above the level of the earth, and thickly covered with mats of rushes and rice-straw, elegantly decorated. These mats are used instead of chairs, and there are no tables, but you will be provided with a little raised tray when you take refreshments. There are no beds—you must sleep upon mats, sit upon mats, smoke upon mats, and fidget upon mats.

Observe that the rooms are separated by folding screens of gilt or colored papers, and lighted by windows of oiled paper, for glass is unknown. You cannot warm yourself at the fire—there is, alas! no fireplace; but in the middle of the room you may crouch down on the brink of the square-tiled hole, from which ascend the fumes of charcoal. The said charcoal, by-the-by, is always burning, and over it a kettle of hot water is always boiling. The Japanese drink tea as voraciously as English old women; but they use little sugar; don't put many spoonfulls into the pot, and serve it up in porcelain cups.

The bath-room resembles European bath-rooms in its general appointments; but it is more frequently resorted to than in our chilly British Isles. The Japanese men bathe, the woman bathe, the children bathe, in-doors and out of doors, morning, noon, and night. The water movement is universal, and most zealously followed out.

At the top of the house is a large tub of water, as a resource in the not unfrequent event of a conflagratian. No London insurance company, we fancy, would insure, at any premium, the inflammable structures of bamboos, screens, oiled papers, mats, and timber ycleped by the Japanese—*houses.* There are wooden tanks in the streets, and rude fire-engines at appointed stations—where the alarm is given by the patrols, who on discovering the first shooting flames, strike forcibly the thick planks, suspended from posts for that purpose.

The Japanese women, according to recent travellers, are models of amiability and good temper, graceful in their manners and attractive in their persons. But they dye their lips a fierce scarlet, their cheeks a violet, and stain their teeth black, with a detestable gangrenous compound—practices scarcely in harmony with the toilet artifices of an English belle. They are fond of dress, of course, or would they be women?

The Japanese gentleman is, generally, a well-looking, intelligent, and active individual. He wears two swords—a large and a small one; while the middle class man is only entitled to one sword; and "the lower orders" carry none. He carries a fan wherever he goes, and whatever he does, and he delights in huge trousers, like a sheet "stitched up between the legs, though open at the sides, in order to allow of the play of the feet while walking." His shoes, and his horse's shoes are made of plaited straw. Consequently, they wear out with unequalled rapidity, and force upon their wearer a shambling, shuffling gait, like Robinson's in the "Wandering Minstrel." Tanners and curriers are not in good odor in Japan, for they have to touch the bodies of the dead—a necessity which the Japanese religion, singularly enough, resents.

Rendall, in his "Memorials of the Empire of Japan," pronounces an opinion on the Japanese character which seems admirably impartial:—"They carry," he says, "their notions of honor to the verge of fanaticism, and they are haughty, vindictive and licentious. On the other hand, brawlers, braggarts, and backbiters are held in the most supreme contempt. The slightest infraction of truth is punished with severity; they are open-hearted, hospitable, and as friends, faithful to death. It is represented that there is no peril a Japanese will not encounter to serve a friend; that no torture will compel him to betray a trust; and that even the stranger who seeks aid will be protected to the last drop of blood."—*London Journal.*

